# Healthcare efficiency assessment using DEA analysis in the Slovak Republic

**DOI:** 10.1186/s13561-018-0191-9

**Published:** 2018-03-09

**Authors:** Robert Stefko, Beata Gavurova, Kristina Kocisova

**Affiliations:** 1 Faculty of Management, The University of Presov, Presov, Slovakia; 2 Faculty of Economics, Technical University of Kosice, Kosice, Slovakia

**Keywords:** Healthcare system, Healthcare technical efficiency, Data envelopment analysis, Healthcare facility, Regional disparity

## Abstract

A regional disparity is becoming increasingly important growth constraint. Policy makers need quantitative knowledge to design effective and targeted policies. In this paper, the regional efficiency of healthcare facilities in Slovakia is measured (2008–2015) using data envelopment analysis (DEA). The DEA is the dominant approach to assessing the efficiency of the healthcare system but also other economic areas. In this study, the window approach is introduced as an extension to the basic DEA models to evaluate healthcare technical efficiency in individual regions and quantify the basic regional disparities and discrepancies. The window DEA method was chosen since it leads to increased discrimination on results especially when applied to small samples and it enables year-by-year comparisons of the results. Two stable inputs (number of beds, number of medical staff), three variable inputs (number of all medical equipment, number of magnetic resonance (MR) devices, number of computed tomography (CT) devices) and two stable outputs (use of beds, average nursing time) were chosen as production variable in an output-oriented 4-year window DEA model for the assessment of technical efficiency in 8 regions. The database was made available from the National Health Information Center and the Slovak Statistical Office, as well as from the online databases Slovstat and DataCube. The aim of the paper is to quantify the impact of the non-standard Data Envelopment Analysis (DEA) variables as the use of medical technologies (MR, CT) on the results of the assessment of the efficiency of the healthcare facilities and their adequacy in the evaluation of the monitored processes. The results of the analysis have shown that there is an indirect dependence between the values of the variables over time and the results of the estimated efficiency in all regions. The regions that had low values of the variables over time achieved a high degree of efficiency and vice versa. Interesting knowledge was that the gradual addition of variables number of MR, number of CT and number of medical devices together, to the input side did not have a significant impact on the overall estimated efficiency of healthcare facilities.

## Background

The issue of healthcare in its broader concept falls within the public sector. The public sector is often viewed in terms of the efficient use of public resources. In the Slovak Republic [23, 24, 62], but also in other countries, several studies have been carried out to evaluate the efficiency of public organizations, including hospital facilities [26, 47, 58]. Potential users of performance measurement results in the healthcare system are governments, regulators, healthcare providers, and the general public. The significant systemic complexity of the healthcare sector determines the analytical level of research studies and the choice of adequate methods for assessing the efficiency of the healthcare sector. Their application is influenced by both the research objectives and the available database. Due to significant systemic changes in the healthcare sector related to the ongoing source of diagnosis and treatment processes, these changes should also be reflected in the outputs of these processes (increasing efficiency in the sector). Some methods may be sensitive to the implementation of so-called non-standard variables, in others the influence is minimal. The research question is to determine to what extent the standard application of methods using classical variables is sufficient for national and international comparison of the efficiency of the surveyed units. In the context of the above, the goals of our contribution were set, with the result and process line. The primary objective of the contribution was to evaluate healthcare technical efficiency in individual regions on the basis of the theoretical backgrounds, practical experience and secondary research on the current state of healthcare facilities in Slovakia and quantify the basic regional disparities and discrepancies. The secondary objective is to quantify the impact of the non-standard variables in DEA related to the use of medical technologies (MR, CT) on the results of the evaluation of the efficiency of the healthcare facilities and their adequacy in the evaluation of the monitored processes. To evaluate the technical efficiency of healthcare facilities at the regional level, we used the extended DEA window analysis, under the conditions of constant (CCR model) as well as the variable return to scale (BCC model). The database was made available from the National Health Information Center and the Slovak Statistical Office, as well as from the online databases Slovstat and DataCube. The input and output variables have been compiled on the basis of a detailed analysis of the most commonly used variables in research studies and taking into account the theoretical rules for the construction of DEA models and limitations for sample size determination.

### Data envelopment analysis and its analytical and application aspects in healthcare

DEA window analysis has been used by several types of research over the past two decades. Its particular application in the healthcare sector can be found in studies of Kazley and Ozcan [33] or Jia and Yuan [31]. In Slovakia, the issue of healthcare and the application of the DEA window analysis method were dealt with by Sendek et al. [60], who focused on assessing the efficiency of hospitals in the Czech and Slovak Republics using the BCC model. Many authors prefer the application of DEA methods due to several advantages like simultaneous use of multiple inputs and outputs (e.g. [13, 16, 27, 28, 34]), it does not require a mathematical specification of the production function (e.g. [13, 27, 28, 50]), it is most appropriate to investigate the impact of exogenous variables [32], suggests recommendations for an inefficient production unit [49]. On the other hand, its application spectrum is eliminated by several disadvantages, resp. limitations. The most important are results are sensitive to outlier values (e.g. [29, 45, 49, 57, 66]), it’s just about measuring relative efficiency [28, 49, 56], limitation for the sample size [38].When choosing a DEA model, it is necessary to define initially if the input or output-oriented method will be used. The used methods are different for application to the healthcare sector. The output-oriented method is preferred by Araújo et al. [2], Hernandez and San Sebastian [28], Oikonomou et al. [51], Li and Dong [38], Karagiannis [32], Cheng et al. [13], Villalobos et al. [66]; Mujasi et al. [49], Mahate et al. [45], De Souza et al. [18]. The input-oriented method was used in studies presented by Grosskopf et al. [27], Kontodimopoulos et al. [34], Czypionka et al. [16], Rezapour et al. [57], Büchner et al. [10], Fragkiadakis et al. [22]. Opinions on DEA and its benefits vary. Hernandez and San Sebastian [28] stated that in the case of primary and secondary healthcare, inputs are uniform and low, and health outcomes could increase at efforts to achieve improved health promotion. They also point out that in many cases the needs for healthcare services are poorly met. In such situations, it would be unethical to reduce the amount of provided healthcare services to improve hospital efficiency. Cheng et al. [13] justify the choice of output orientation due to limited control of hospital managers over their inputs and controlled decisions on recruitment and investment by government departments. Oikonomou et al. [51] justified the choice of an output-oriented model because the demand for primary health care services has a tendency to expand and not to decrease. According to these authors, lowering inputs in the provision of health services is undesirable, while increasing outputs is feasible. Based on the study of literature, we decided to use an output-oriented model in our analysis, as the primary objective in the field of healthcare is the human health. When questioning hospital efficiency, it is important to focus attention on the quality of the services provided, on the quantity and satisfaction of patients and to focus on increasing patient satisfaction due to a better and better healthcare system. This will result in more treatment, more performance, more hospitalization, more release, and, as a result, an increase in quality of life and health, decreasing levels of disease mortality, late diagnosis and inadequate treatment. From a moral point of view, the healthcare system is specific and the aim of hospitals and healthcare facilities should not be to reduce inputs and costs but to concentrate on increasing outputs in the form of the above-mentioned objectives. For this reason, we prefer to use an output-oriented DEA model. The second important theoretical decision in DEA specification is the application of return to scale. The model assuming the constant return to scale was defined by Charnes et al. [12] and is marked as the CCR model. Second, the model assuming with the variable return to scale was described by Banker et al. [3] and is labelled as BCC. In the context of the defined goals, we decided to apply both approaches and compare the results achieved through both types of models.

## Methods

Data Envelopment Analysis (DEA) is the dominant non-parametric approach to evaluate the efficiency of Decision-Making Units (DMU). From the point of view of assessing the efficiency of healthcare, DMUs can represent different levels of healthcare, including a complete healthcare system in the country, districts, hospitals, specific service providers, departments, or individual physicians. The efficiency of the DMU represents its distance from the efficiency frontier. The location and shape of the efficiency frontier depend on the user data and the used assumptions (a type of return to scale, input or output orientation). The efficiency frontier arises by plotting the relationship between the number of inputs and the outputs achieved in the two-dimensional space. This is a combination of individual tracking relationships between inputs and outputs. The construction of the efficiency frontier is based on the principle of the best estimate, so we only regard it as an approaching reality [15]. From a historical point of view, we consider Farrell and his work in 1957 to lay the foundations of the DEA method. Farrell [21], in his work, starts to measure efficiency by assuming that only one input enters the model and production unit produces only one output. However, as the authors of the first comprehensive model are considered Charnes et al. [12], who extended the original Farrell’s model. This basic model is referred to by the initials of its authors, the CCR model, assuming that the production unit operates under the conditions of constant return to scale. The second basic model was developed by Banker et al. [3], named as the BCC model and is based on the assumption of the variable return to scale.

In the healthcare sector, there is imperfect competition, which is manifested by limited funding opportunities, market entry regulation, merger or market exit constraints resulting in inefficient management, so it is necessary to apply the BCC model in addition to the CCR model. DEA models can be input, or output oriented. While in the case of input-oriented models we try to find out the minimal level of inputs which is needed to produce a given level of outputs. Under the output-oriented models, we try to find out the answer which maximum level of outputs can be achieved by using the given level of inputs in order for DMUs to be considered effective.

In order to evaluate the technical efficiency of the healthcare system at the level regions of the Slovak Republic, we decided to apply the output-oriented models, CCR and BCC, based on the DEA window analysis. Output oriented CCR model can be formulated in the matrix form by the following formula:

1 $$ {\displaystyle \begin{array}{l}\operatorname{Maximize}\kern9em g={\phi}_q+\varepsilon \left({e}^T{s}^{+}+{e}^T{s}^{-}\right)\\ {}\mathrm{Under}\ \mathrm{conditions}\kern3.5em X\lambda +{s}^{-}={x}_q\kern1em \\ {}\kern15.5em Y\lambda -{s}^{+}={\phi}_q{y}_q\kern1em \\ {}\kern15.74em \lambda, {s}^{+},{s}^{-}\ge 0\end{array}} $$

*where: ε – constant, q – evaluated DMU, y*_*q*_ *– output of evaluated DMUq, x*_*q*_ *– input of evaluated DMUq, s* ^+^ *and s* ^−^ *are slack variables for input and outputs.*

As stated by Jablonský and Dlouhý [30] based on the model (1), the production unit is evaluated as effective if the optimal value of the function g* = 1 and all complementary variables are equal to zero. If this value is above 1, the DMU cannot be considered as an efficient and the optimal value ${\phi}_q^{\ast }$ expresses the need for a proportional increase in inputs to achieve efficiency. Assuming that production units operate under the variable return to scale (increasing, decreasing, non-increasing, non-decreasing), we apply the BCC output-oriented model, where we add a convexity condition: *e*^*T*^*λ* = 1.

When evaluating efficiency, we can sometimes encounter a limited number of DMUs. To overcome this problem, a so-called DEA window analysis was created. It allows us to compare the efficiency of a limited number of DMUs in individual periods and to analyze changes in efficiency over time. DEA window analysis generalizes the idea of moving averages to uncover the trend of DMU efficiency development over time. The moving average method is used to compile a different sample to determine the relative efficiency of each DMU. Based on the dynamic perspective, each DMU is considered as a separate unit in individual time periods in individual windows [15]. The input and output variables of DMU in the selected period are compared to that of other DMUs in all periods. We also compare the results of DMU from one period with the results of the same unit in the remaining periods. If the window is moved for the first time, at the same time the first period is deleted in each window and a new period is added. The benefit of this method is a comprehensive description of dynamic changes of the efficiency of each DMU, both horizontal and vertical. Of course, the main benefit is what we mentioned in the beginning, increasing the number of DMUs, which increases the discriminatory power in situations with a limited number of DMUs in the sample [31].

We assume a sample *N* (*n* = 1,...,*N*) DMUs during *T* (*t* = 1,...,*T*) periods of time. Each DMU uses *r* different inputs to produces *s* different outputs. If DMU_n_^t^ is a combination of inputs and outputs for the *N*th unit of the DMU in the *T* period, then the input vector *X*_*n*_^*t*^ and output vector *Y*_*n*_^*t*^ can be written as follows:

2 $$ {X}_n^t=\left[\begin{array}{c}{x}_n^{1t}\\ {}\vdots \\ {}{x}_n^{rt}\end{array}\right]\kern0.5em {Y}_n^t=\left[\begin{array}{c}{y}_n^{1t}\\ {}\vdots \\ {}{y}_n^{st}\end{array}\right]\kern1em $$

If the window starts in time *k* (1 ≤ *k* ≤ *T*) and the width of the window is *w* (1 ≤ *k* ≤ *T*-*k*), then input matrix (*X*_*k*_^*w*^) and output matrix (*Y*_*k*_^*w*^) of each window will look like as follows:

3 $$ {X}_k^w=\left[\begin{array}{cc}{x}_1^k& {x}_2^k\\ {}\vdots & \vdots \\ {}{x}_1^{k+w}& {x}_2^{k+w}\end{array}\kern1em \begin{array}{cc}\cdots & {x}_N^k\\ {}\ddots & \vdots \\ {}\cdots & {x}_N^{k+w}\end{array}\right]\kern0.5em {Y}_k^w=\left[\begin{array}{cc}{y}_1^k& {y}_2^k\\ {}\vdots & \vdots \\ {}{y}_1^{k+w}& {y}_2^{k+w}\end{array}\kern1em \begin{array}{cc}\cdots & {y}_N^k\\ {}\ddots & \vdots \\ {}\cdots & {y}_N^{k+w}\end{array}\right]\kern0.5em $$

Appropriate determination of window size and window length are used by Cooper et al. [15] where we use the following labels: *n = number of DMU, k = number of periods, p = window length (p ≤ k), w = number of windows.* The following relationships apply to:

number of windows:

4 $$ w=k-p+1 $$

number of DMU in each window:

5 $$ np/2 $$

number of different DMUs:

6 $$ npw $$

Δ number of DMU (increase in number):

7 $$ n\left(p-1\right)\left(k-P\right) $$

To derive the total number of different DMUs, Cooper et al. [15] mention the following relationship:

8 $$ n\left(k-p+1\right)p $$

If we put the last equation equal to 0, we get a relationship for calculating the length of window:

9 $$ p=\frac{k+1}{2} $$

To always reach an integer, the relationship applies:

10 $$ p=\left\{\begin{array}{c}\frac{k+1}{2}\kern2.75em , when\ k\  is\  odd;\\ {}\frac{k+1}{2}\pm \frac{1}{2}\kern2em , when\ k\  is\ even.\end{array}\right. $$

In this paper, we will analyze the Slovak health care system. We have decided to monitor the DMU at the regional level as the best comparable minimum level of tracking. The smaller division into districts was not taken into account due to the lack of availability of microdata at the relevant regional level. The indicators monitored by multinational organizations are used as the smallest regional level of NUTS 3, which is the breakdown in the region when applying to Slovakia. In Slovakia, we have a total of eight districts. In the first step, DEA window analysis is performed to calculate the technical efficiency of healthcare facilities in the relevant regions. The selection of the DEA analysis is influenced by a number of sources that use the DEA method to assess the efficiency of medical devices. A review of the literature using the DEA method can be seen in the work of Worthington [68].

Due to the frequent criticism of the DEA method which is based on its nonparametric nature and is mirrored in a small statistical basis, one of the most important steps is the right choice of input and output variables. After the study of relevant literature, we decide to use five input and three output variables in our analysis. The specification of the variables used in the DEA, their summary overview and the brief definition are given in Table 1.

**Table 1:** Specification of DEA model variables

Labels	Variable	Definition
Input variables		
x1	Number of beds	Total number of beds in the health facility
x2	Number of medical staff	Total number of medical staff, including the number of physicians and nurses
x3	Number of CT	Number of computed tomography (CT) devices
x4	Number of MR	Number of magnetic resonance (MR) devices
x5	Number of medical equipment together	Number of all medical devices
Output variables		
y1	Bed occupancy rate	Percentage use of the total number of beds
y2	Average nursing time in days	Ratio of treatment days to the total number of hospitalized patients

Source: Prepared by authors

The number of beds is an indicator that reflects the size of the hospital. It is clear from this indicator that each added bed means an extra cost to the hospital for its purchase and future operation. On the other hand, the beds mean the possibility of providing basic hospital services, thus bringing the marginal profit to the hospital. Whether directly from the patient or from health insurance companies that reimburse hospitals for payments made for medications, consumed other medical supplies besides drugs themselves. The number of beds is one of the most commonly used indicators for comparing hospitals across the country. This fact was put forward by Wagstaff [67] in his study in the evaluation of Spanish hospitals in 1977–1981. Ley [37] had a sample of 139 Spanish hospitals, Valdmanis [64] compared US hospitals in Michigan, Byrnes and Valdmanis [8] compared the sample of 123 Californian hospitals, Kooreman [35] analysed nursing houses in the Netherlands, Zuckerman et al. [69] compared an extensive sample to 4149 American hospitals, Lopez-Valcarcel and Perez [42] compared the efficiency of Spanish hospitals in 1991–1993 with the DEA and Stochastic Frontier Approach (SFA) method; Magnussen [44] evaluated the efficiency of Norwegian hospitals, Parkin and Hollingsworth [52] evaluated hospitals in Scotland, Linna [39] compared the results of the efficiency of the SFA, DEA and Malmquist methods on a sample of 43 Finnish acute care hospitals, Burgess and Wilson [9] compared 1545 US hospitals using DEA, Linna et al. [40] followed a sample of 48 Finnish acute care hospitals, Gerdtham et al. [25] compared 26 Swedish hospitals using SFA, and Maniadakis and Thanassoulis [46] analyzed the efficiency of 75 Scottish hospitals for a 5 year period. Several authors used a variable number of beds for international comparisons, such as Varabyova and Schreyögg [65], who compared OECD countries to each other. Dervaux et al. [17] compared the French and American hospitals, Samut and Cafri [59] compared 29 OECD countries between 2000 and 2010, Mobley and Magnussen [48] were using the DEA analysis to compare public, highly regulated Norwegian hospitals with a private, highly competitive unregulated system hospitals in California, USA.

The number of the medical staff represents the registered number of employees in natural persons, being the sum of the number of doctors, dentists, pharmacists, nurses, midwives, laboratory technicians, assistants, technicians and other health workers. With the authors Maestre et al. [43], Baray and Cliquet [4], we see the tracking of the basic indicator as the total number of employees without subdivision of employees into subgroups. According to these authors, the total number of employees is the basic indicator needed to monitor the economic outturn. The indicator also reflects the size of the patient’s catchment area, which according to the geographical location of the hospital is used by the particular hospital [43]. Employee tracking alone has no telling value. Of course, the number of employees is largely affected by the increase in wage costs, with each additional employee in employment [4]. An indicator of the total number of employees was used in the studies by Byrnes and Valdmanis [8], Kooreman [35], Lopez-Valcarcel and Perez [42], Magnussen [44], Parkin and Hollingsworth [52], Maniadakis and Thanassoulis [46]. The total number of medical personnel, other technical personnel, and the number of non-medical personnel was used as an input variable by Cheng et al. [13] who used variable numbers of doctors, nurses, and the number of administrative and other staff. Czypionka et al. [16] used the variable of medical and non-medical staff, Varabyova and Schreyögg [65], and Li and Dong [38] used the variable of employees for the DEA model, Mahate et al. [45] distributed input variables to physicians, dentists, nurses, pharmacists, administrative and other workers to estimate technical efficiency. Fragkiadakis et al. [22] used inputs such as clinical staff, nurses and administrative staff, Rezapour et al. [57] used the total staff of hospitals in the aggregate variable: human resources. Blank and Valdmanis [6] used a variable to describe the efficiency of hospitals: employees and administrators, nurses, medical staff, other staff. Variable medical equipment denounces the overall technical equipment of hospitals and medical facilities and is expressed in pieces. In particular, we monitor the total number of technical devices including radiograph, mammography, positron tomograph, linear accelerators, electroencephalographs, ultrasound devices, brachytherapy devices, urethroscopes, bronchoscopes, endoscopes, laparoscopes, arthroscopes, tomographs, angiography, monitoring devices, ultraviolet and infrared emitters, colposcopes, laryngoscopes and pharyngoscopes, dialysis monitors, gamma cameras, isotopic irradiators, electromyographs, agnetic resonance, lithotriptors, cytoscopes, colonoscopes, sigmoidoscopes and rectoscopes, gastroscopes and duodenoscopes, cryogenic devices. Particularly, we focus on variable magnetic resonance (MR) and computed tomography (CT) devices, as they are the most widely used devices for the diagnosis of diseases and injuries in the human body. Some authors have used variable medical devices as a whole, like Grosskopf et al. [27] who used as inputs assets all buildings and facilities. Zuckerman et al. [69] used as an input variable the index of high-tech services. Puig-Junoy [54] used as one of the input variables the technological availability measured as a proportion of 33 technological items available in the intensive care unit expressed as a percentage. Bradford et al. [7] used the variable use of established and new technology. Dey et al. [19] used as a variable “capital facilities”, defined as the provision of up-to-date technological equipment and equipment, also referred to in the second subcategory “maintenance” defined as reasonable and regular maintenance of medical equipment by the biomedical team of engineers. Chang and Lan [11] studied the impact of using new technologies on early diagnosis and treatment, Tsekouras et al. [63] monitored the impact of purchasing new high-tech medical devices to increase production efficiency, Kounetas and Papathanassopoulos [36] monitored the efficiency of Greek hospitals and the impact on efficiency by introducing advanced medical facilities, Rezaee and Karimdadi [56] used the input medical device, Oikonomou et al. [51] used the biomedical technology variable, which it divided by weight into a fully operational microbiological and imaging laboratory and a group of twenty diagnostic therapeutic and auxiliary devices, Ancarani et al. [1] examined the impact of the acquisition of relevant medical technologies and information technologies on the efficiency of hospital departments in three state hospitals in Dubai. The impact of the introduction of information technology on the efficiency of the health system was also examined by Šoltés et al. [61]. Another group of authors focused on monitoring only individual MR or CT devices: Lo et al. [41], who monitored the effect of the MR, CT, röntgen, Chirikos and Sear [14] variable scan devices, used composite indices to measure efficiency, reflecting special MR tests and procedures. Retzlaff-Roberts et al. [55] used the number of MR devices per million population to confirm the growth and necessity of healthcare technologies, and Samut and Cafri [59] used input variables to count the MR and CT devices per 100,000 inhabitants.

Bed occupation is used by many authors in their studies, Kooreman [35], Linna [39], Chirikos and Sear [14], Perera et al. [53], Belciug and Gorunescu [5], Dy et al. [20], Rezaee and Karimdadi [56], Rezapour et al. [57]. An indicator of the use of the bed fund generally refers to the percentage utilization of the total number of hospital beds for a specified period, typically a calendar year [5]. As noted by Dy et al. [20], this indicator directly reflects the use of resources available to the hospital. Too low the value of the use of bedding is a warning signal for inefficient use of financial resources and hospital capacities, which should lead to a reduction in the number of beds with unchanged patient satisfaction, but to lower the cost of bed operation and maintenance [53]. The number of beds is directly related to the ownership of immovable property. Finding the optimal size of the use of bedding is key to effective hospital management. The Average Length of Stay (ALOS) tells you the length of the patient’s length on the bed in the facility. It is calculated as a proportion of the total number of treatment days and the number of hospitalized patients. If the average treatment time would be reduced, the total costs would be reduced as the cost of treating the patient on the bed would be reduced. Also, the trend of lower-cost outpatient treatment is also reduced in even more complicated cases. On the other hand, there is a presumption that shorter stays are more demanding and therefore expensive. If the patient is discharged too quickly and healing is not possible, re-hospitalization may occur, which may even increase the cost. The variable average treatment time in days was used by Kooreman [35], Chirikos and Sear [14], Varabyova and Schreyögg, [65] to investigate the efficiency of DEA patients.

## Results and discussion

The analysis was done using a narrower sample of years under review for the period 2008 to 2015. The reason for narrowing the sample is the consistency of data on medical devices and specific MR and CT devices reported since 2008. Therefore it was not able to use data since 2000. The total number of DMUs is 8 regions in each year (8 years). As the number of observations is only 56, which is a relatively low number, we decided to apply the DEA window analysis to expand the number of DMU units. The length of individual windows being calculated based on assumptions and relationships defined in the methodology. Altogether, eight models have been formulated. The following table shows the assignment of selected variables to defined models (Table 2).

**Table 2:** Specification of DEA models

Variables	m1	m2	m3	m4	m5	m6	m7	m8
Input variables								
x1	X	X	X	X	X	X	X	X
x2	X	X	X	X	X	X	X	X
x3					X	X		
x4			X	X				
x5							X	X
Output variables								
y1	X	X	X	X	X	X	X	X
y2	X	X	X	X	X	X	X	X

*Notes* m1, m3, m5, m7 - CCR models; m2, m4, m6, m8 - BCC models; X - symbolizes that the variable is contained in model; x1 - number of beds; x2 - number of medical staff; x3 - number of CT devices; x4 - number of MR devices; x5 - the number of all medical devices; y1 – bed occupancy rate; y2 - average nursing time in days

Source: Prepared by authors

For the calculation of the efficiency, data were collected for the reference period from 2008 to 2015. A summary of the basic descriptive statistics of the variables for each year separately and globally for the whole analysed period is shown in Table 3. From the descriptive statistics, we can see that in the case of the variable “number of beds” the maximum is approximately up to two times higher compared to a minimum in each year. This suggests that the size of the regional distribution is significant and the results in regions are different up to twofold when comparing the minimum and maximum in the sample. A similar but even more pronounced difference across regions can be seen in the variable “number of medical staff” where the maximum for the whole surveyed period is up to 2.87 times higher compared to the minimum. The biggest differences across the reporting period were recorded in two districts: Bratislava region (maximum) and Trnava region (minimum).

**Table 3:** Summary descriptive statistics of variables in calculating efficiency using DEA

		Variables						
		x1	x2	y1	y2	x3	x4	x5
2015	Min	2437	6022	221	7	7	2	813
	Max	5381	17,299	263	9	15	9	1720
	Average	3934	10,040	244	8	11	5	1149
	Median	3945	9264	249	8	11	4	1098
2014	Min	2408	6202	218	7	7	2	813
	Max	5554	17,248	267	8	15	9	1720
	Average	3952	9966	245	8	11	5	1149
	Median	3934	8995	248	8	11	4	1098
2013	Min	2373	6134	223	7	6	1	787
	Max	5563	17,054	264	8	15	10	2100
	Average	3954	9933	245	8	10	4	1182
	Median	3956	8787	251	8	10	3	1214
2012	Min	2348	6120	224	7	6	0	746
	Max	5356	17,127	268	8	15	9	2055
	Average	4030	9904	246	8	10	4	1277
	Median	3931	9056	249	8	10	3	1165
2011	Min	2533	6246	214	7	6	0	674
	Max	5736	17,163	263	9	16	11	1999
	Average	4119	9855	237	8	10	5	1232
	Median	3954	8790	242	8	9	3	1114
2010	Min	2637	6424	215	7	6	0	666
	Max	5934	16,472	258	9	18	11	1954
	Average	4392	9944	238	8	9	5	1196
	Median	4233	9040	243	8	8	4	1086
2009	Min	2568	6483	217	7	5	0	581
	Max	5988	16,031	251	9	13	10	1901
	Average	4440	9745	238	8	9	4	1122
	Median	4326	8977	241	9	9	5	1056
2008	Min	2558	6513	221	8	4	0	571
	Max	5930	15,405	251	9	14	10	2163
	Average	4460	9892	239	8	9	4	1100
	Median	4285	9539	242	9	9	5	974
2008–2015	Min	2348	6022	214	7	4	0	571
	Max	5988	17,299	268	9	18	11	2163
	Average	4162	9898	242	8	10	4	1193
	Median	4078	9475	244	8	9	4	1118

*Notes* x1 - number of beds in pieces; x2 - number of medical staff in persons; x3 - number of CT devices; x4 - number of MR devices; x5 - the number of all medical devices; y1 – beds occupancy in days; y2 - average nursing time in days

Source: Own calculations

The variable “beds occupancy in days” is less volatile across the region compared to the previous two. For the whole analysed period the maximum is only 1.25 times higher compared to a minimum. The average minimum bed occupancy is 242 days per year, with a minimum value of 214 days and a maximum of 268. The most productive was the Nitra region in 2012 with a total of 268.4 days per year. The worst result of only 214.0 days per year was reached by the Trnava region in 2011. The average daily “nursing time” across all regions for the whole of the monitored period was declining, so the regions reduced the caregiving time for the reference period 2008 to 2015. The highest average nursing times were in Kosice region in 2008 and the Nitra region in two consecutive periods 2009 and 2010. The shortest nursing time was 6.8 days in the Trnava region in 2014 and 2015. For the indicator, the number of MR devices, the differences can be again marked across regions. The lowest number of MR devices was recorded in the Trencín region in 2008 - 2012 and the highest number in the Bratislava region in 2011. The average in all regions is 4 MR devices. The number of CT devices is l higher compared to MR ones. The average over the monitored period is 10 CT devices. The most CT devices were in Bratislava in 2010 with a total of 18 CTs. The variable total number of medical equipment brings the largest difference between the maximum value of 2163 devices in the Bratislava region and the minimum of 571 devices in the Trencín region, both in 2008. As regards the median values of the variables in the monitored period, the following situations occurred. The number of beds fell from 4285 to 3945, a decrease of 8%. The number of health workers dropped from 9539 to 9264, a decrease of 3%. The use of beds in days increased from 242 to 249 days, an increase of 3%. Average nursing time in days decreased from 9 to 8, a decrease of 11%. The number of CT devices has increased from 9 to 11, which is a 22% increase. A drop from 5 to four MR devices represents a 20% drop. And finally, the number of medical equipment has risen from 974 units to 1098 units, an increase of 13%.

Estimated efficiencies for the years 2008–2015 using DEA analysis of different models are shown in Table 4. According to the m1 model, the Trnava, Trencin, Nitra and Banska Bystrica regions reached average efficiencies above the average of the whole sample. Below the average, there are the remaining regions, namely Zilina, Bratislava, Presov and Kosice region. The efficiency score equal to one can be seen in the Trnava region in 2008, 2011, 2012 and 2015. The Trencin region is efficient in 2011, 2012 and 2015. The third region that achieved efficiency equal to 1 is the Nitra region in 2009. The best average values reached the Trnava region with the value of 0.9929. On the other hand, the minimum average value was achieved in Kosice region (0.5262). The Kosice region recorded the biggest change, decreased by 13.74%. Besides Kosice region, the Zilina region (10.80%) and the Presov region (9.29%) also fell. The largest increase was recorded in the Banska Bystrica region (5.73%).

**Table 4:** Estimation of the efficiency of the DEA model average for whole analysed period (2008–2015) according different models

	m1	m2	m3	m4	m5	m6	m7	m8
Bratislava	0.5635	0.9618	0.5635	0.9618	0.5764	0.9618	0.5635	0.9618
Trnava	0.9929	0.9955	0.9947	0.9974	0.9983	1.0000	0.9954	0.9967
Trencin	0.9898	0.9922	0.9904	1.0000	0.9973	0.9982	0.9974	0.9986
Nitra	0.9427	0.9983	0.9430	0.9983	0.9713	0.9986	0.9653	0.9985
Zilina	0.6963	0.9325	0.6971	0.9436	0.6991	0.9325	0.6963	0.9325
Banska Bystrica	0.8545	0.9501	0.8563	0.9534	0.8546	0.9501	0.8558	0.9501
Presov	0.6651	0.9137	0.6661	0.9137	0.6716	0.9137	0.6728	0.9137
Kosice	0.5262	0.9553	0.5303	0.9605	0.6653	0.9557	0.5622	0.9553
Average	0.7789	0.9624	0.7802	0.9661	0.8042	0.9638	0.7886	0.9634

Source: Prepared by authors

The m2 model compared to the m1 model reached higher average efficiency (0.9624). The average efficiencies above the average were also achieved by the Trencin and Trnava regions, as in the m1 model. Also, the Nitra region achieves efficiency at level 1, namely in 2009–2012, 2014 and 2015. According to the model m2, the Banska Bystrica, Zilina, Presov and Kosice region are located below the average. The most efficient were the Trnava, Trencin and Nitra regions with only minor deviations. Efficiency qual to one was reached by Trnava region in 2011, 2012 and 2015; Trencin region during the period 2011–2015; Nitra region during the period 2009–2012, 2014 and 2015 and Kosice region in 2008. The Trencin and Nitra regions were growing and the declines were reached in Bratislava, Zilina, Banska Bystrica, Presov and Kosice regions, with the biggest drop of Kosice region by 6.15%.

Model m3 has an average value of estimated efficiency over the whole reference period at 0.7802, which is significantly less than in the model m2 and more than the model m1. Fully efficient units were: Trnava region in 2008, 2011, 2012 and 2015; Trencin region in 2011,2012 and 2015; Nitra region in 2009. Above average were: Trencin, Trnava, Nitra and Banska Bystrica regions. Below the average were: the Zilina, Bratislava, Presov and Kosice regions. The increase in efficiency was recorded by the Bratislava, Trencin, Nitra and Banska Bystrica regions. The decline was recorded in Zilina, Presov and Kosice regions. The biggest loss of efficiency was reached by Zilina region, with a decrease around 11%. The most effective unit was the Trnava region with an average efficiency of 0.9947, while the least efficient unit was the Kosice region with an average efficiency of 0.5303.

The average value in model m4 of all regions for the whole surveyed period is 0.9661 and this results in the top three m1, m2 and m3 models. This is the highest average estimated efficiency. Above average there were Trencin, Trnava and Nitra regions. The Banska Bystrica region is no longer above the average of the Slovak Republic, but below it, along with the Zilina, Kosice and Presov region. For the first time, the model set the Trencin region as effective in every monitored year from 2008 to 2015. For this reason, the estimated value for the whole period for the Trencín region equals 1. In the Trnava region, as in the Trencin region, during the monitored period, there is zero change of efficiency. The declines were recorded in the Bratislava, Zilina, Banska Bystrica, Presov and Kosice regions. The most significant fall was recorded in the Zilina region with a total decrease of 7.34%.

The fifth is the m5, where the total average of the estimated efficiency for all regions is 0.8042 with a slight overall increase of 0.82%. Above average results, there were traditionally four regions, the same as in other previous models, namely Trencin, Trnava, Nitra and Banska Bystrica region. Under the average were Zilina, Presov, and Kosice regions. The highest average efficiency was reported by the m5 model in 2009, where the average efficiency in this year was 0.8130. On the other hand, the lowest efficiency was estimated on average for 2008 with average efficiency equal to 0.7783. The lowest efficiency is reached in the Bratislava region (0.5764). The highest performance on average was reached the Trnava region with an efficiency of 0.9983 without significant changes across the time period. An increase in the estimated efficiency was achieved by Bratislava, Banska Bystrica and Kosice regions. Kosice region recorded the most significant growth of 18.34%. The decline was recorded by the Nitra, Zilina and Presov regions, where the largest loss was in the Presov region (9.81%).

The m6 model, on average, has an estimated efficiency of 0.9638 across the whole reference period, with a slight decrease of 2.11%. The most effective unit with an estimated efficiency of 1 is Trnava region in 2008, 2010–2015. Another one is Trencin region in 2008, 2009, 2011–2015, and Nitra region with an efficiency of 1 in 2009–2015, and Kosice region in 2008. Above average were the Trnava, Trencin and Nitra regions. Below the average, there are Banska Bystrica, Zilina and Presov region. The decline in efficiency was in the Bratislava region by a slight 0.91%, in the Zilina region by 2.75%, in the Banska Bystrica by 2.06% in Presov by 6.43% and in Kosice region by 6.01%. The growth was only in one region, namely 1.12% in the Nitra region.

The average value of efficiency estimated by the m7 model for the whole period from 2008 to 2015 for the whole of Slovakia is 0.7886 with a total decline of 0.32%. As in all previous models, the above-average were Trnava, Trencin, Nitra and Banska Bystrica regions. Below the average were Zilina, Presov, Bratislava and Kosice regions. Efficiency equal to 1 was achieved by the Trnava region in 2008, 2011, 2012 and 2015; Trencin region in 2008, 2011, 2012 and 2015; and Nitra region in 2009. The increase in average efficiency for the whole analysed period was reached by the Bratislava region (3.68%), Nitra region (0.49%), Banska Bystrica region (6.75%) and Kosice region (12.70%). On the other side, the decline was recorded in the Zilina region (10,80%) and Presov region (12.82%).

The last model in the model group was the model m8. This model estimates the overall average efficiency for all regions over the observed period of 0.963394 with a fall of 2.13%. The Trencin, Trnava and Nitra regions are efficient throughout the whole period under review. Kosice region is efficient only in 2008. The increase in efficiency was reached by Nitra region (1.12%). In the other regions, there was a decline in efficiency, namely in the Bratislava region by 0.91%, in the Zilina region by 2.75%, in the Banska Bystrica region by 2.06%, in the Presov region by 6.43% and in the Kosice region by 6.15%.

After performing efficiency estimates using 8 models and partial analyzes each of them, we can conclude that every model created produces very similar results, no model speculated a special extreme value that would be inconsistent with other models. Therefore, we can conclude that the results shown and described in the previous sections are consistent with regard to the choice of input variables and roughly equally estimate the efficiency and inefficiency of Slovak regions. We note that the gradual substitution of the variables “number of MR devices ”, “number of CT devices” and “number of all medical devices ” to input side did not significantly affect the overall estimated efficiency of healthcare services in regions. Based on these findings, we consider that impact of selected input variables: × 1 - the number of beds and  x2 - the number medical staff, and selected outputs: y1 – bed occupancy rate and y2 - average nursing time, was significant, and therefore the adding other variables affected the overall efficiency only in minimal way. The selection of the variables x1, x2, y1 and y2 is crucial for the evaluation of the efficiency of healthcare devices and we have confirmed this in the analysis. In the process of evaluation of the impact of medical technology (apparatus and equipment) on the efficiency, we recommend to add inputs or outputs which are directly results of the usage of medical technology in the field of treatment or prevention. In all models of DEA analysis, the regions of Trencin, Trnava and Nitra were repeated above the average. Banska Bystrica region was completing them, which was just above the average. In the m2, m4, m6 and m8 they were complemented by the Kosice region, but only in 2008, at the beginning of the monitored period and then gradually in each of the aforementioned models, the efficiency of the Kosice region had fallen over time. When we look at the development of variables, we repeat ourselves in the overriding values of the opposite of the other regions. Above average of whole sample during the whole analysed period, ie the highest average values, were at the  x1 - Bratislava, Kosice and Presov regions. For variable  x2 - the regions of Bratislava, Kosice, Presov and Zilina were above the total average. For variable y1 - the regions of Zilina, Kosice, Bratislava, Nitra and Banska Bystrica were above the overall average for this variable; y2 - regions of Bratislava, Nitra, Presov, Kosice and Banska Bystrica were above the y2 overall average; x3- Banska Bystrica,  Bratislava and Zilina were above the total average; x4- Bratislava, Kosice, and Presov. were above the average; and for the last variable x5 the regions of Bratislava, Zilina and Kosice were above the average of whole sample for this variable. We see that Trencin and Trnava regions are not above the average in any input and output variables. What is important is whether they can provide adequate bad occupancy rate and average nursing time in days with the number of beds and the number of medical staff. In the deeper analysis and comparison of the number of beds and the use of beds, we can see that the regions with the highest number of beds are using them less than regions with fewer beds and therefore it is a clear recommendation to reduce the number of beds in Bratislava, Kosice, Zilina and Presov regions in order to increase the use of beds, as these regions have large surpluses of beds, resp., could increase the number of hospitals and thus their use. Another way is to increase their occupancy with unchanged bed counts. When looking at the ratio of the number of medical staff to the second observed variable “average nursing time in days”, we get similar results as in the previous situation. In the Bratislava, Kosice and Presov regions, the proportion is too high, suggesting an optimal reduction in the number of medical staff. The same result is obtained by the proportional variations in the number of medical staff in the variable use of beds. Recommendations based on performance analysis are for Bratislava, Kosice, Zilina and Presov regions in terms of output orientations for increasing the use of beds with unchanged bed counts and the number of health workers. It also comes to our belief that the average time of care/treatment should be increased for Bratislava, Kosice, Zilina and Presov with unchanged beds and medical staff. This outcome is debatable, since increasing the average treatment time may mean making more demanding treatment procedures, hospitalization of which is demanding, which is desirable in terms of demand for health services. There is a tendency to reduce the average treatment time due to the shift of hospitalized performance to outpatient care and thus to the treatment of beds and the working time of healthcare workers. Unregulated reductions in average treatment time may lead to re-hospitalizations and re-operations, which reduces efficiency.

## Conclusion

In the context of the ongoing globalization processes and pressures on the efficiency of healthcare systems in the countries, our study focused on analyzing and evaluating the efficiency of healthcare facilities in the various regions of the Slovak Republic in order to detect significant disparities. We applied the DEA method, used in many research studies to evaluate the efficiency of the sectors. Estimated efficiencies did not differ significantly in our models, but were consistent regardless of the change in input variables. The results of the analysis have shown that there is an indirect dependence between the values of the variables over time and the results of the estimated efficiency in all regions. The regions that had low values of the variables over time achieved a high degree of efficiency and vice versa. Interesting knowledge was that the gradual addition of variables “number of MR devices ”, “number of CT devices ” and “number of all medical devices ” to the input side did not have a significant impact on the overall estimated efficiency of healthcare facilities. Technological advances in recent decades have brought many new diagnostic and therapeutic devices and healthcare practices that are also costly and which are expected to have significant health and economic benefits. For this reason, these processes need to be taken into account in systems for measuring and evaluating the efficiency of healthcare facilities. Our DEA method has not uncovered the impact of health technologies on health care efficiency, which significantly reduces its importance in the process of quantifying and evaluating efficiency in the future. In Slovakia, unlike in the rest of the world, the use of Health Technology Assessment (HTA) is totally absent. The efficiency of outputs from HTA is significantly increased by complementary use of adequate efficiency measurement methods, which would also take into account disparities and disproportions in the use of new health technologies in healthcare units on an aggregate scale. It is only possible to explicitly quantify the health and economic outcomes of their use and to set up a platform for national and international benchmarking. These specific facts encourage the realization of subsequent research aimed at finding, resp. designing appropriate systems to measure the efficiency of the healthcare system and their process interconnection. Their importance is particularly important in the process of creating stabilization and regulatory mechanisms in the health system and in the development of targeted policies.
